# Non-iron Deficiency Anemia in Rural Indian Women: A Cross-Sectional Study

**DOI:** 10.7759/cureus.28565

**Published:** 2022-08-29

**Authors:** Somen Saha, Tapasvi Puwar, Komal Shah, Apurvakumar Pandya, Mayur B Wanjari, Deepak Saxena

**Affiliations:** 1 Public Health, Indian Institute of Public Health Gandhinagar, Gandhinagar, IND; 2 Public Health, Jawaharlal Nehru Medical College, Datta Meghe Institute of Medical Sciences (Deemed to be University), Wardha, IND; 3 Community Medicine, Indian Institute of Public Health Gandhinagar, Gandhinagar, IND; 4 Public Health, Indian Institute of Public Health (IIPH) Gandhinagar, Ahmedabad, IND; 5 Epidemiology and Public Health, Parul Institute of Public Health, Parul University, Vadodara, IND; 6 Research and Development, Jawaharlal Nehru Medical College, Datta Meghe Institute of Medical Sciences (Deemed to be University), Wardha, IND; 7 School of Epidemiology and Public Health, Jawaharlal Nehru Medical College, Datta Meghe Institute of Medical Sciences (Deemed to be University), Wardha, IND

**Keywords:** india, hemoglobin, ferritin, non-iron deficiency anemia, iron-deficiency anemia

## Abstract

Introduction

Anemia is an important public health concern, affecting almost 25% of the global population. In India, these statistics are even more worrisome with more than half of the children, non-pregnant and pregnant women being affected by the disease. Though the major cause of anemia is iron deficiency, other causes cannot be neglected considering the magnitude of the problem. The present study was designed to estimate the prevalence of non-iron deficiency anemia (NIDA) in Devbhoomi Dwarka District of Gujarat.

Methods

For this cross-sectional study, total of 258 mothers (antenatal and postnatal) were enrolled from 27 primary health centers. Apart from demographic details and obstetric history, hemoglobin and ferritin levels were measured from the participant’s blood after obtaining consent. Clinical information such as the history of recent illness and fever were also noted through questionnaires. Prevalence was anemia was calculated using a standard threshold of blood hemoglobin and NIDM was defined using ferritin levels.

Results

As per hemoglobin levels, anemia was present in 65.9% of the mothers, which was relatively higher in antenatal women (90%) as compared to postnatal women (57%). Ferritin levels showed that out of this total anemic mothers, 61.8% (105/258) have normal ferritin levels indicating the presence of non-iron deficiency. Trimester-wise analysis of anemia in AN showed that NIDM prevalence increases with increasing trimester in contrast to IDA where a considerable reduction was found with iron supplementation.

Conclusion

The study indicated that two out of every three women were anemic and only one out of four were anemic with depleted iron storage. These findings have important policy implications as in India the anemia control programs address only iron deficiency anemia and not NIDA. The development of a framework for diagnosis and appropriate treatment can be recommended for integration into national guidelines.

## Introduction

Anemia is a major public health concern in India with 58.6% of children, 53.2% of non-pregnant women, and 50.4% of pregnant women being affected by the condition. Though the condition has consequences throughout the life course of an individual during pregnancy it may lead to serious complications [1].

Various studies have documented Iron deficiencies are the most common cause of anemia in the world [2]. Albeit the share of iron deficiency in overall anemia statistics varies and depends on numerous population-specific factors. Other well-known contributors to anemia are 1) micronutrient deficiencies such as folate, B12 and vitamin A; 2) infections such as malaria, TB and worm infestation; 3) hemorrhoids; 4) other inherited diseases of hemoglobin synthesis [3]. Blood hemoglobin estimation is a widely used method to diagnose anemia in all age groups. However, studies have shown that it provides a crude estimation of the iron concentration as iron is only a part of hemoglobin and its level cannot be accurately correlated with the total iron level. For estimating total iron storage other biomarkers such as ferritin and transferrin are more reliable.

Anemia prevention and control in India is “Iron deficiency centric” where under the Intensive National Iron Plus Initiative (INIPI), [4] oral and parenteral iron preparation, folate and albendazole are emphasized for the prevention and treatment of maternal anemia. As per current protocol, screening and management of maternal anemia are planned by hemoglobin (Hb) estimation [4] only, and treatment is being given irrespective of the status of iron as well as other micronutrient storage [5,6]. Also, food fortification and food diversity are promoted as a strategy to prevent nutritional anemia. Albeit in spite of these efforts of the last three decades the anemia prevalence has not shown much improvement in India. This could be partially due to the multifactorial nature of the condition where not only iron but also other deficiencies are also playing a significant role [7-10].

Existing literature on iron-deficiency anemia (IDA) and non-iron deficiency anemia (NIDA) are in the global context; however, the prevalence of both in the Indian population is sporadically reported. The present study was designed to assess the prevalence of NIDA in antenatal (AN) and prenatal (PN) mothers from Devbhoomi Dwarka District of Gujarat State, India. We also aim to correlate it with the overall anemia burden in this subset of the population.

This article was previously posted to the medRxiv preprint server on February 12, 2021.

## Materials and methods

In a cross-sectional study, a total of 258 pregnant AN and PN women were enrolled in 27 primary health centers. Anemia was assessed based on hemoglobin concentration obtained from venous whole blood, using an auto-analyzer. Iron storage of the participants was assessed by measuring serum ferritin concentration. Direct chemiluminescence method using MINI VIDAS, which is a compact automated immunoassay system based on the Enzyme-Linked Fluorescent Assay (ELFA) principles were used to estimate ferritin levels. 

The pregnant women of any trimester (AN women) and PN women within 90 days of pregnancy were included in the present study after obtaining written consent. Sociodemographic and clinical details of participants such as name, age, gender, residence and obstetric and illness history were collected through pre-tested semi-structured questionnaires. For estimation of biochemical parameters (hemoglobin and ferritin), 4 mL of blood was drawn from every participant 4 mL by a phlebotomist and lab technicians, at the respective primary health centers following standard procedure. The specimens were transported to the laboratory within one hour of collection and were stored at -20°C until the time of analysis.

Anemia was defined as hemoglobin <11% in AN women and <12% in PN women [5]. IDA was defined as a combination of anemia and ID while NIDA is defined as the presence of anemia without ID. Low serum ferritin without a history of fever was considered a biomarker for iron deficiency. Any women with signs or symptoms of acute inflammation in the last seven days were excluded from the study.

Data were analyzed using SPSS version 22 (IBM Corp., Armonk, NY). Descriptive analysis was performed where categorical variables were expressed as numbers and percentages. Formal approval was obtained from the District Authority and Ethical clearance was sought from the Institutional Ethics Committee at the Indian Institute of Public Health Gandhinagar.

Ethical consideration

The study subjects sought written consent, and formal approval was obtained from state and district authorities. The Institutional Ethics Committee of the Indian Institute of Public Health, Gandhinagar, approved the study (IEC/IRB approval number 14/2019-20).

## Results

Mean age of the study participants was 25.3 years, which was almost similar to the group of AN and PN women (25.7 and 25.1 years respectively). Overall, the prevalence of anemia (low hemoglobin) in the study population was 65.9%. The burden of anemia was greater in AN (90%) as compared to PN (57%), though it could not reach a statistically significant level due to the smaller sample size. The prevalence of IDA was slightly higher in AN (40.8% vs 30%). Trimester-wise analysis showed that there is a steep increase in anemia prevalence with the trimesters, however, there was a reduction in ID (Table 1). Detailed analysis showed that the increase in total anemia was contributed by a corresponding increase in NDIA (Table 1).

**Table 1:** Trimester-wise prevalence of overall anemia, iron-deficiency and non-iron deficiency anemia

Trimester	Total anemia, N (%)	Iron-deficiency anemia, N (%)	Non-iron deficiency anemia, N (%)
First	42	50	50
Second	80	40.7	59.3
Third	66.3	39.7	60.3

## Discussion

To the best of our knowledge, this is the first Western Indian study providing the prevalence of NIDA in rural mothers. As hypothesized the study results pointed out that the prevalence of NIDA in AN and PN is high and needs to be addressed by anemia-controlled programs. The study has provided two key findings - 1) almost 60% of anemic cases are NIDA and 2) these numbers are not improved with iron-folate supplementation provided during pregnancy.

Our findings were supported by a few global studies as well. About 27.1% of the study participant had ID (low s. ferritin) while 38.2% of them were screened for anemia (low Hb) in the present study which is within the range of 19% to 61% reported in various studies conducted in low and middle-income countries [11]. The prevalence of NIDA was significantly higher than IDA in all participants as well as in AN and PN women groups. In a review of European iron-status studies in women [12], ID was reported in 25%-77% of pregnant women, and IDA was reported in 6-30% of pregnant women. A study conducted among AN women in a tertiary care center in India reported 34% IDA and 14% NIDA [13]. Muthukumaran et al. [14] reported an IDA proportion higher (60%) than the present study (40%) in the third trimester of AN women. The proportion of IDA was lower than NIDA in the second trimester among anemic women. Prophylactic oral iron consumption during the second and third trimesters might be the reason behind this variation.

As per National guidelines, the screening of anemia is being carried out among AN and PN women by hemoglobin estimation while no screening is being carried out for iron deficiency in India. Comprehensive National Nutrition survey 2016-18 (CNNS 2016-18), had reported a higher prevalence of the NIDA (16.9%) than IDA (12%) among adolescents (age 10-19 years) by using s. ferritin as a screening tool and also stated ID as the strongest predictor of anemia among adolescents [15]. Similar to that large-scale survey of AN, as well as PN women by ferritin estimation, can be useful to know the burden of the IDA, as well as NIDA and focus, which can be prioritized for anemia control.

Various preventive measures are being carried out to address nutritional anemia including food fortification and food diversity but for the treatment of anemia especially moderate to severe, oral and parenteral iron along with folate are indicated in the second and third trimesters irrespective of the iron status of the body [4]. Due to physiological depletion in iron storage, iron supplements are mandatory in the pregnancy period [16]. As observed in the present study, a large number of AN mothers (59.2%) were anemic might be due to causes other than the ID [17]. Even globally it was evidenced that reason behind half of the anemic patients was other than ID [2]. Thus, in the context of nutritional anemia, other micronutrient deficiencies needed to be assessed and addressed for tackling maternal anemia effectively.

Limitation of the study

There are several factors that can influence the ferritin level such as inflammation. Any women with signs or symptoms of acute inflammation in the last seven days were excluded from the study. Low serum ferritin without a history of fever was considered a biomarker for iron deficiency. Contributing factors of non-iron deficiencies were not assessed in this study and remain an area of future research.

## Conclusions

IDA is still a public health problem. However, the proportion of NIDA observed in the present study is alarming that ought to be addressed. Two out of every three women are anemic; one out of four were anemic with depleted iron storage. Importantly, two out of five women had anemia but iron storage was sufficient. To achieve the ambitious National nutrition target 2025 - 50% reduction of anemia in women of reproductive age, strategy to prevent and correct anemia must include screening for iron and non-iron deficiency anemia and following appropriate treatment protocol for both types of anemia.
